# Interplay between *PNPLA3* genotype, liver steatosis, and cardiac remodeling in young adults: A magnetic resonance imaging study

**DOI:** 10.14814/phy2.71040

**Published:** 2026-08-07

**Authors:** Nick S. R. Lan, Phillip E. Melton, Amro Sehly, Leon A. Adams, John K. Olynyk, Oyekoya T. Ayonrinde, Brendan Adler, Lawrence J. Beilin, Trevor A. Mori, Girish Dwivedi

**Affiliations:** ^1^ Medical School The University of Western Australia Perth Western Australia Australia; ^2^ Department of Cardiology, Centre of Excellence for Cardiometabolic Health Fiona Stanley Hospital Perth Western Australia Australia; ^3^ APEX Heart Lab Harry Perkins Institute of Medical Research Perth Western Australia Australia; ^4^ Menzies Institute for Medical Research, College of Health and Medicine University of Tasmania Hobart Tasmania Australia; ^5^ School of Global and Population Health The University of Western Australia Perth Western Australia Australia; ^6^ Department of Hepatology Sir Charles Gairdner Hospital Perth Western Australia Australia; ^7^ Curtin Medical Research Institute Curtin University Perth Western Australia Australia; ^8^ Department of Gastroenterology Fiona Stanley Hospital Perth Western Australia Australia; ^9^ Medical School Curtin University Perth Western Australia Australia; ^10^ Envision Medical Imaging Perth Western Australia Australia; ^11^ CardioInnovate Translational Lab Western Australia Victor Chang Cardiac Research Institute Sydney New South Wales Australia

**Keywords:** cardiac, cardiometabolic, genetics, liver, metabolic, MRI

## Abstract

Steatotic liver disease (SLD) is associated with cardiovascular disease. The *PNPLA3* rs738409C>G variant is a genetic determinant of liver fat accumulation. We investigated the association of the *PNPLA3* rs738409C>G variant with cardiac structure and function in young adults, stratified by SLD status. Participants underwent genotyping at 17 years and liver and cardiac magnetic resonance imaging at 27 years. SLD was defined as volumetric liver fat fraction >3.55%. *PNPLA3* rs738409 was analyzed primarily using a dominant genetic model (CC vs. CG/GG). Multivariable linear regression was used to evaluate the association between *PNPLA3* rs738409C>G and cardiac parameters. Of 657 participants, 38.1% carried *PNPLA3* rs738409C>G and 16.4% had SLD. Associations between *PNPLA3* rs738409C>G and cardiac parameters were contingent on SLD status, with significant PNPLA3 × SLD interactions observed for left ventricular mass index (LVMi; *p* = 0.009), left ventricular end‐diastolic volume index (LVEDVi; *p* = 0.015), and right ventricular end‐diastolic volume index (RVEDVi; *p* = 0.049). *PNPLA3* rs738409C>G was associated with greater LVMi (*p* = 0.014), LVEDVi (*p* = 0.005), and RVEDVi (*p* = 0.040) among individuals with SLD, but not without SLD. In this non‐mechanistic observational study, *PNPLA3* rs738409C>G was associated with subclinical cardiac remodeling only in the presence of SLD. These hypothesis‐generating findings and any potential mechanisms require confirmation in future studies.

## Graphical abstract

The *PNPLA3* rs738409 variant was associated with cardiac remodeling, manifested as greater left ventricular mass, left ventricular end‐diastolic volume and right ventricular end‐diastolic volume, among young adults with steatotic liver disease but not in those without steatotic liver disease.

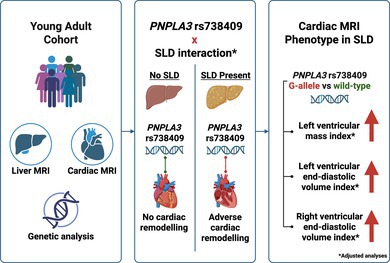

## 1 INTRODUCTION

Steatotic liver disease (SLD) affects 30% of adults globally and encompasses metabolic‐dysfunction associated SLD, alcohol‐related liver disease and mixed phenotypes (Israelsen et al., 2024). SLD is a cardiometabolic condition which increases the risk of atherosclerotic cardiovascular disease (ASCVD) and heart failure (HF) (Huangfu et al., 2023; Israelsen et al., 2024; Møller et al., 2025). However, the risk of cardiac complications in SLD may depend on the underlying mechanism of liver fat accumulation (Ahmed et al., 2024; Jamialahmadi et al., 2024). Genetic variation in the *PNPLA3* (patatin‐like phospholipase domain‐containing protein 3) gene, particularly I148M (rs738409), is the strongest known determinant of metabolic‐dysfunction associated SLD (Luukkonen et al., 2023; Romeo et al., 2008). In some, but not all studies, the *PNPLA3* rs738409 G‐allele has been paradoxically associated with a lower risk of coronary artery disease and myocardial infarction, possibly due to hepatocyte retention of triglycerides and a reduction in circulating lipoproteins (Ahmed et al., 2024; Guo et al., 2025; Jamialahmadi et al., 2024; Wijarnpreecha et al., 2020; Wu et al., 2020). It may be postulated that reduced hepatic secretion of triglycerides results in less cardiac “lipotoxicity,” with reduced lipid accumulation in epicardial adipose tissue or the myocardium (Butcko et al., 2026). However, whether genetically mediated SLD is associated with subclinical alterations in cardiac structure and function from a young age, and therefore long‐term risk of HF, remains unknown (Hydes et al., 2024).

Cardiac magnetic resonance imaging (MRI) provides gold‐standard, non‐invasive assessment of cardiac structure and function (Salerno et al., 2017). Similarly, liver MRI fat fraction provides precise evaluation of liver steatosis (Harry et al., 2020; St Pierre et al., 2016). In this observational study, we investigated the associations between *PNPLA3* genotype, SLD and cardiac remodeling in a well‐characterized cohort of young adults at age 27 years, and specifically assessed whether associations with cardiac structure and function are contingent on the presence of SLD (Dontje et al., 2019; Straker et al., 2017).

## 2 MATERIALS AND METHODS

### 2.1 Study population

The Raine Study (https://rainestudy.org.au/) has been described previously (Dontje et al., 2019; Straker et al., 2017). In brief, 2900 pregnant women were enrolled between May 1989–November 1991 to examine outcomes of frequent antenatal ultrasounds (Dontje et al., 2019; Straker et al., 2017). Subsequently, 2868 offspring were prospectively followed from birth (Dontje et al., 2019; Straker et al., 2017). As the study is voluntary and due to attrition (e.g., deaths, lost to follow‐up due to relocation away from Western Australia and inability to participate due to time commitments), 1150 offspring completed questionnaires, 1082 provided physical assessments, 1060 underwent fasting blood tests, and 975 had liver and cardiac MRI at age 27 years. Genetic (17 years), liver MRI (27 years), and cardiac MRI (27 years) data were available for 657 participants for the current analysis. This cohort is broadly representative of the wider Australian population at this age (Straker et al., 2017). Comparison of the study cohort (*n* = 657) with the remainder of participants with physical assessments or blood tests at age 27 years found no significant difference in gender, body mass index, waist circumference, exercise (metabolic equivalents per week), history of dyslipidaemia, hypertension or diabetes, blood pressure, and lipid profile (*p* > 0.05); however, the study cohort was more likely to be Caucasian (96.0% vs. 77.2%; *p* < 0.001). The Raine Study was approved by the University of Western Australia Human Research Ethics Committee, is registered in the Australian New Zealand Clinical Trials Registry (ACTRN12617001599369), and was conducted according to the principles of the Declaration of Helsinki. Participants gave informed written consent.

### 2.2 Genotyping

DNA was extracted from whole blood using a Puregene DNA isolation kit at the 17‐year assessment in consenting participants. Genotyping was performed on an Illumina BeadArray Reader with the Illumina Human660‐W Quad Array and imputation using MACH v.1.0.16 against a reference of the North and Western European genetic ancestry samples of HapMap phase 2, build 36, release 22, as previously described. (Hudert et al., 2022). Quantitative polymerase chain reaction using the TaqMan assay (Thermo Fisher #4351379) was performed to determine rs738409C>G in *PNPLA3* (Hudert et al., 2022).

### 2.3 Magnetic resonance imaging

Liver and cardiac MRI was performed at a single centre (Envision Medical Imaging, Perth, WA, Australia) with a 1.5‐T scanner (Siemens Magnetom Aera 1.5T, Erlangen, Germany) using a body coil (32‐channel). Liver steatosis was quantified using a validated volumetric liver fat fraction equation (HepaFat) which measures the volume fraction of the liver tissue occupied by fatty vesicles and has been validated and approved by the Food and Drug Administration for the quantification of liver steatosis (Harry et al., 2020; St Pierre et al., 2016). SLD was defined as a volumetric liver fat fraction >3.55%, which has been demonstrated to have a very high degree of accuracy (area under curve 0.945–0.960), sensitivity (91.4%–96.7%) and specificity (91.4%–92.3%) for the determination of hepatic steatosis determined by liver biopsy (i.e., grade 0 vs. 1–3), by two independent liver pathologists (Harry et al., 2020; St Pierre et al., 2016).

Cardiac MRI was evaluated using Segment CMR software (Medviso AB, Lund, Sweden) by blinded readers supervised by a level 3 expert reader. Electrocardiographic gating was performed according to recommended protocols (Kramer et al., 2020). Standard recommended imaging planes were used to measure left ventricular (LV) and right ventricular (RV) volumes and function, LV mass and left atrial volumes (Kramer et al., 2020). Measurements were indexed to body surface area where relevant. Intraclass correlation coefficients showed very good intra‐observer variability for key cardiac MRI parameters (LV mass index 0.988, LV ejection fraction 0.897, LV global longitudinal strain 0.984) as previously described (Sehly et al., 2025).

### 2.4 Statistical analyses

Continuous variables are presented as mean ± standard deviation (normally distributed data) or median with interquartile range (skewed data), whilst categorical variables are presented as number (percent). Characteristics were compared across groups using Student's *t*‐test, Mann–Whitney *U* test, one‐way analysis of variance or Kruskal‐Wallis test for continuous variables, and Pearson's chi‐square test or Fisher's exact test for categorical variables. Increased alcohol intake was defined as >20 g/day for females and >30 g/day for males (Israelsen et al., 2024). *PNPLA3* rs738409 was analyzed primarily using a dominant genetic model (CC vs. CG/GG) and secondarily using an additive genetic model (0, 1 or 2 copies of the G‐allele) (Romeo et al., 2008) rather than a recessive genetic model (CC/CG vs. GG) due to low number of homozygotes (*n* = 44). Hardy–Weinberg equilibrium (HWE) was assessed in the subgroup without SLD, consistent with standard practice in case–control genetic studies, because genotype frequencies in cases or combined samples may deviate from equilibrium when a variant is associated with disease.

The primary analysis assessed effect modification by SLD using an interaction term (PNPLA3 × SLD) in sex‐adjusted multivariable linear regression models. Cardiac parameters with a pre‐specified interaction *p* < 0.10 were carried forward into stratified analyses to avoid missing potentially meaningful effect modification in these exploratory analyses. Correction for multiple comparisons was not performed due to the hypothesis‐generating nature of these analyses focused on interaction effects. In stratified models, *PNPLA3* rs738409 was examined within SLD and non‐SLD subgroups, with *β* coefficients and 95% confidence intervals presented separately. These models were adjusted for sex, waist circumference, hypertension, systolic blood pressure, homeostatic model assessment of insulin resistance [HOMA‐IR; fasting insulin (μU/L) × fasting glucose (nmol/L)/22.5] and alcohol intake at age 27 years, reflecting characteristics at the time of the MRI scans. Sensitivity analyses were performed adjusting for body mass index instead of waist circumference and in participants with SLD, MRI liver fat fraction as a continuous variable. Analyses were performed in SPSS Statistics (v29, IBM, Armonk, NY, USA). A *p* < 0.05 was considered statistically significant.

## 3 RESULTS

Of 657 participants, 325 (49.5%) were male, 250 (38.1%) were carriers of rs738409C>G in *PNPLA3* and 108 (16.4%) had SLD. Participant characteristics are shown in Table 1 according to rs738409C>G in *PNPLA3* carrier status and SLD status. Individuals carrying the *PNPLA3* rs738409 G‐allele had a significantly lower body mass index (*p* = 0.049) and a higher MRI liver fat fraction (*p* = 0.007) compared with non‐carriers. Characteristics by number of *PNPLA3* rs738409 G‐alleles (CC: 61.9%; CG: 31.4%; GG: 6.7%) are shown in Table 2; a significant difference between groups was observed for MRI liver fat fraction (*p* = 0.003). HWE p‐values for all groups were: full cohort *p* = 0.013; SLD *p* = 0.011; without SLD (CC: 62.7%; CG: 31.7%; GG: 5.6%) *p* = 0.154. Deviation from HWE in the full cohort likely reflects enrichment of the variant among individuals with SLD.

**TABLE 1:** Baseline characteristics of study population by rs738409C>G in *PNPLA3* carrier status and steatotic liver disease.

Characteristic	*PNPLA3* wild‐type carrier (*n* = 407)	*PNPLA3* rs738409 G‐allele carrier (*n* = 250)	*p*‐value	No SLD (*n* = 549)	SLD^a^ (*n* = 108)	*p*‐value
Male sex	203 (49.9%)	122 (48.8%)	0.789	262 (47.7%)	63 (58.3%)	**0.044**
Height (cm)	173 (167–182)	174 (167–181)	0.793	173 (167–181)	176.5 (168–183)	0.082
Weight (kg)	76 (66–88)	74 (65–86)	0.164	73 (64–84)	99 (82–116)	**<0.001**
BMI (kg/m^2^)	24.8 (22.6–28.1)	23.8 (22.1–27.5)	**0.049**	23.9 (21.9–26.5)	31.1 (26.9–36.4)	**<0.001**
BSA (m^2^)	1.9 (1.8–2.1)	1.9 (1.7–2.1)	0.274	1.9 (1.7–2.0)	2.2 (2.0–2.4)	**<0.001**
Waist circumference (cm)	82.3 (75.1–91.7)	81.7 (74.0–91.2)	0.441	80.6 (73.5–88.0)	100.9 (89.6–113.7)	**<0.001**
Caucasian ethnicity	394 (96.8%)	237 (94.8%)	0.200	526 (95.8%)	105 (97.2%)	0.786
Paternal cardiac history	3 (0.8%)	3 (1.4%)	0.677	5 (1.0%)	1 (1.1%)	0.999
Maternal cardiac history	12 (3.2%)	7 (3.0%)	0.906	18 (3.5%)	1 (1.0%)	0.339
Smoker	62 (15.7%)	44 (18.2%)	0.406	93 (17.4%)	13 (12.4%)	0.202
Dyslipidaemia	7 (1.8%)	3 (1.3%)	0.750	7 (1.4%)	3 (3.0%)	0.215
Hypertension^b^	11 (2.9%)	2 (0.9%)	0.146	6 (1.2%)	7 (6.9%)	**<0.001**
Systolic BP (mmHg)	123.7 ± 13.0	122.0 ± 12.4	0.114	121.7 ± 11.8	130.0 ± 15.1	**<0.001**
Diastolic BP (mmHg)	75.4 ± 8.2	74.6 ± 8.2	0.236	74.1 ± 7.7	80.7 ± 8.7	**<0.001**
Diabetes	2 (0.5%)	1 (0.4%)	0.999	3 (0.6%)	0	0.999
HOMA‐IR	1.1 (0.8–1.6)	1.1 (0.8–1.6)	0.432	1.0 (0.8–1.4)	2.3 (1.7–2.9)	**<0.001**
Total cholesterol (mmol/L)	4.8 (4.2–5.3)	4.8 (4.2–5.4)	0.964	4.7 (4.2–5.3)	5.1 (4.2–5.6)	**0.023**
LDL‐C (mmol/L)	2.9 ± 0.7	2.9 ± 0.7	0.961	2.8 ± 0.7	3.1 ± 0.8	**<0.001**
HDL‐C (mmol/L)	1.4 (1.2–1.7)	1.4 (1.2–1.7)	0.600	1.5 (1.2–1.7)	1.2 (1.0–1.3)	**<0.001**
Triglycerides (mmol/L)	0.9 (0.7–1.2)	0.9 (0.7–1.2)	0.794	0.8 (0.6–1.1)	1.3 (0.9–1.8)	**<0.001**
Alcohol (g/day)	6.6 (1.6–17.9)	8.1 (1.5–21.6)	0.360	7.5 (1.9–18.5)	3.9 (0.6–20.7)	0.122
Exercise (MET/week)	1900 (600–3732)	2232 (658–4500)	0.258	2097 (692–4038)	1404 (310–4158)	0.240
MRI liver fat fraction (%)	1.5 (1.1–2.3)	1.7 (1.2–2.6)	**0.007**	1.4 (1.1–1.9)	7.0 (5.2–13.1)	‐

*Note*: Data are presented as number (percentage), median (quartile 1–3) or mean ± standard deviation. Bold values indicate values of *p* < 0.05.

Abbreviations: BMI body mass index; BP blood pressure; BSA body surface area; HDL‐C high‐density lipoprotein cholesterol; HOMA‐IR Homeostatic Model Assessment for Insulin Resistance; LDL‐C low‐density lipoprotein cholesterol; MET metabolic equivalents; MRI magnetic resonance imaging; *PNPLA3* patatin‐like phospholipase domain‐containing protein 3; SLD steatotic liver disease.

^a^ Diagnosis based on MRI volumetric liver fat fraction >3.55%.

^b^ Defined as systolic blood pressure >140 mmHg and/or diastolic blood pressure >90 mmHg based on average rested readings.

**TABLE 2:** Baseline characteristics of study population by *PNPLA3* genotype.

Characteristic	*PNPLA3* rs738409 (CC) (*n* = 407)	*PNPLA3* rs738409 (CG) (*n* = 206)	*PNPLA3* rs738409 (GG) (*n* = 44)	*p*‐value
Male sex	203 (49.9%)	103 (50.0%)	18 (43.2%)	0.689
Height (cm)	173 (167–182)	174 (167–182)	173.5 (167–179)	0.719
Weight (kg)	76 (66–88)	73.5 (65–86)	76.5 (63–88)	0.379
BMI (kg/m^2^)	24.8 (22.6–28.1)	23.8 (22.1–27.4)	23.7 (22.0–28.7)	0.138
BSA (m^2^)	1.9 (1.8–2.1)	1.9 (1.7–2.1)	1.9 (1.7–2.1)	0.541
Waist circumference (cm)	82.3 (75.1–91.7)	82.0 (74.2–91.3)	80.8 (73.5–93.6)	0.706
Caucasian ethnicity	394 (96.8%)	197 (95.6%)	40 (90.9%)	0.126
Paternal cardiac history	3 (0.8%)	3 (1.7%)	0	0.609
Maternal cardiac history	12 (3.2%)	5 (2.6%)	2 (4.9%)	0.649
Smoker	62 (15.7%)	37 (18.4%)	7 (17.1%)	0.692
Dyslipidaemia	7 (1.8%)	2 (1.0%)	1 (2.4%)	0.557
Hypertension^a^	11 (2.9%)	1 (0.5%)	1 (2.4%)	0.135
Systolic BP (mmHg)	123.7 ± 13.0	122.5 ± 12.8	119.8 ± 10.7	0.132
Diastolic BP (mmHg)	75.4 ± 8.2	74.7 ± 8.6	74.5 ± 6.6	0.493
Diabetes	2 (0.5%)	1 (0.5%)	0	0.999
HOMA‐IR	1.1 (0.8–1.6)	1.1 (0.8–1.5)	1.1 (0.8–1.7)	0.572
Total cholesterol (mmol/L)	4.8 (4.2–5.3)	4.8 (4.3–5.4)	4.6 (4.0–5.4)	0.578
LDL‐C (mmol/L)	2.9 ± 0.7	2.9 ± 0.7	2.9 ± 0.9	0.954
HDL‐C (mmol/L)	1.4 (1.2–1.7)	1.4 (1.2–1.7)	1.4 (1.3–1.7)	0.594
Triglycerides (mmol/L)	0.9 (0.7–1.2)	0.9 (0.7–1.2)	0.9 (0.7–1.1)	0.966
Alcohol (g/day)	6.6 (1.6–17.9)	8.6 (1.5–20.5)	7.2 (2.4–22.5)	0.657
Exercise (MET/week)	1900 (600–3732)	2130 (615–4250)	2862 (946–5541)	0.231
MRI liver fat fraction (%)	1.5 (1.1–2.3)	1.6 (1.1–2.4)	2.0 (1.3–5.6)	**0.003**

*Note*: Data are presented as number (percentage), median (quartile 1–3) or mean ± standard deviation. Bold values indicate values of *p* < 0.05.

Abbreviations: BMI body mass index; BP blood pressure; BSA body surface area; HDL‐C high‐density lipoprotein cholesterol; HOMA‐IR Homeostatic Model Assessment for Insulin Resistance; LDL‐C low‐density lipoprotein cholesterol; MET metabolic equivalents; MRI magnetic resonance imaging; *PNPLA3* patatin‐like phospholipase domain‐containing protein 3.

^a^ Defined as systolic blood pressure >140 mmHg and/or diastolic blood pressure >90 mmHg based on average rested readings.

Individuals with SLD were significantly more likely to be male (*p* = 0.044) and hypertensive (*p* < 0.001), and have higher weight (*p* < 0.001), body mass index (*p* < 0.001), body surface area (*p* < 0.001), waist circumference (*p* < 0.001), systolic blood pressure (*p* < 0.001), diastolic blood pressure (*p* < 0.001), HOMA‐IR (*p* < 0.001), total cholesterol (*p* = 0.023), low‐density lipoprotein cholesterol (*p* < 0.001), and triglycerides (*p* < 0.001) compared with those without SLD (Table 1). Sex‐stratified analyses of individuals with versus without SLD demonstrated similar findings (Table S1). In participants with SLD, 19 (17.6%) reported increased alcohol intake, of whom 8 (42.1%) were carriers of rs738409C>G in *PNPLA3*.

In the overall cohort, rs738409C>G in *PNPLA3* was not associated in sex‐adjusted analyses with key cardiac MRI parameters (Table S2). However, significant PNPLA3 × SLD interactions were observed for LV mass index (*p* = 0.009), LV end‐diastolic volume index (*p* = 0.015) and RV end‐diastolic volume index (*p* = 0.049) in the dominant model (CC vs. CG/GG). There was no significant interaction for LV end‐systolic volume index (*p* = 0.055) and LV stroke volume index (*p* = 0.052). In the additive model, a significant PNPLA3 × SLD interaction for LV end‐systolic volume index (*p* = 0.038) was also observed but there was no significant interaction for RV end‐diastolic volume index (*p* = 0.083).

Among individuals with SLD, rs738409C>G in *PNPLA3* was associated with greater LV mass index (*β* 3.29; *p* = 0.014), LV end‐diastolic volume index (*β* 6.29; *p* = 0.005), RV end‐diastolic volume index (*β* 5.99; *p* = 0.040), and LV stroke volume index (*β* 4.16; *p* = 0.004) in adjusted models, whereas no significant associations were detected among those without SLD (Table 3). In the additive model, similar associations were observed (Table 3), but there was no significant difference in RV end‐diastolic volume index (*p* = 0.125). Findings were similar in sensitivity analyses adjusting for body mass index (Table S3). Sensitivity analyses adjusting for MRI liver fat fraction (Table S4) also demonstrated similar findings, although associations for RV end‐diastolic volume index (*β* 5.82; *p* = 0.065) were not significant in the primary analysis with the dominant genetic model. Sex‐stratified values for cardiac MRI parameters are shown in Table S5 for those with SLD.

**TABLE 3:** Association between *PNPLA3* genotype and steatotic liver disease on cardiac remodeling.

Parameter	Interaction *p*‐value for PNPLA3xSLD^a^	No SLD (*n* = 549)				SLD^a^ (*n* = 108)			
		*β* (95% CI)^b^	*p*‐value	*β* (95% CI)^c^	*p*‐value	*β* (95% CI)^b^	*p*‐value	*β* (95% CI)^c^	*p*‐value
Dominant genetic model (CC vs. CG/GG)									
LV mass index (g/m^2^)	**0.009**	−0.51 (−1.62–0.60)	0.370	−0.22 (−1.35–0.92)	0.707	3.07 (0.82–5.31)	**0.008**	3.29 (0.68–5.90)	**0.014**
LV EDVi (ml/m^2^)	**0.015**	−0.32 (−2.29–1.65)	0.749	−0.21 (−2.17–1.76)	0.835	5.49 (1.18–9.80)	**0.013**	6.29 (1.97–10.61)	**0.005**
LV ESVi (ml/m^2^)	0.055	−0.28 (−1.43–0.86)	0.628	−0.39 (−1.57–0.78)	0.511	2.33 (−0.27–4.94)	0.079	2.13 (−0.85–5.11)	0.159
LV SVi (ml/m^2^)	0.052	−0.04 (−1.37–1.29)	0.955	0.18 (−1.15–1.52)	0.786	3.16 (0.34–5.97)	**0.028**	4.16 (1.39–6.93)	**0.004**
RV EDVi (ml/m^2^)	**0.049**	−0.60 (−2.97–1.76)	0.615	−1.10 (−3.42–1.21)	0.349	5.15 (−0.06–10.36)	0.053	5.99 (0.28–11.69)	**0.040**
Additive genetic model (0, 1 or 2 copies of the G‐allele)									
LV mass index (g/m^2^)	**0.017**	−0.63 (−1.53–026)	0.166	−0.38 (−1.30–0.55)	0.423	1.77 (0.15–3.39)	**0.033**	2.10 (0.25–3.94)	**0.026**
LV EDVi (ml/m^2^)	**0.014**	−0.41 (−2.00–1.19)	0.615	−0.22 (−1.82–1.38)	0.786	3.86 (0.79–6.92)	**0.014**	4.25 (1.20–7.29)	**0.005**
LV ESVi (ml/m^2^)	**0.038**	−0.36 (−1.29–0.57)	0.445	−0.45 (−1.41–0.50)	0.354	1.67 (−0.18–3.53)	0.076	1.50 (−0.60–3.59)	0.159
LV SVi (ml/m^2^)	0.065	−0.05 (−1.13–1.03)	0.931	0.23 (−0.86–1.32)	0.677	2.18 (0.18–4.19)	**0.033**	2.75 (0.79–4.71)	**0.006**
RV EDVi (ml/m^2^)	0.083	−0.56 (−2.47–1.35)	0.566	−0.93 (−2.81–0.95)	0.333	3.12 (−0.60–6.85)	0.099	3.15 (−0.90–7.20)	0.125

*Note*: Bold values indicate values of *p* < 0.05.

Abbreviations: CI confidence interval; EDVi end‐diastolic volume index; ESVi end‐systolic volume index; HOMA‐IR Homeostatic Model Assessment for Insulin Resistance; LV left ventricular; MRI magnetic resonance imaging; *PNPLA3* patatin‐like phospholipase domain‐containing protein 3; RV right ventricular; SLD steatotic liver disease; SVi stroke volume index.

^a^ Diagnosis based on MRI volumetric liver fat fraction >3.55%.

^b^ Adjusted for sex.

^c^ Adjusted for sex, waist circumference, hypertension, systolic blood pressure, HOMA‐IR, and alcohol intake.

## 4 DISCUSSION

**FIGURE 1:**
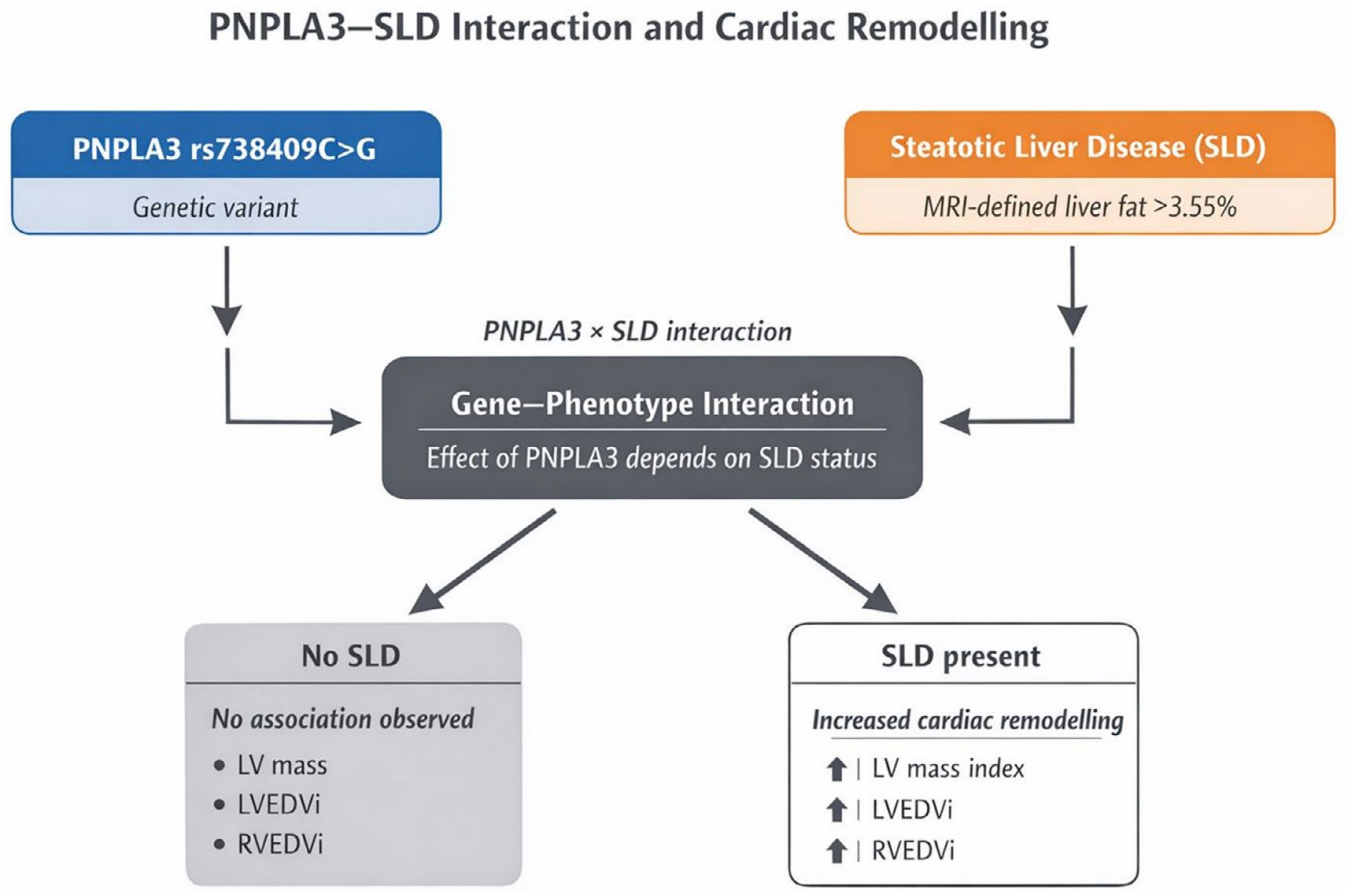
Schematic diagram of PNPLA3‐SLD interaction and cardiac remodeling. LV, left ventricular; LVEDVi, left ventricular end‐diastolic volume index; MRI, magnetic resonance imaging; PNPLA3, patatin‐like phospholipase domain‐containing protein 3; RVEDVi, right ventricular end‐diastolic volume index; SLD, steatotic liver disease.

In this observational cohort study of young adults, rs738409C>G in *PNPLA3* was associated with greater liver fat accumulation, consistent with its established role in SLD. The key finding is that the association between rs738409C>G and cardiac remodeling was contingent on the presence of SLD with greater LV mass, LV end‐diastolic volumes, and RV end‐diastolic volumes among individuals with SLD, but not in those without SLD. This was an observational non‐mechanistic study and does not support *PNPLA3* as an independent causal driver of cardiac remodeling, but that an association may be present and conditional on the liver phenotype (Figure 1). Importantly, myocardial hypertrophy and ventricular dilatation were observed, and these are known precursors of adverse cardiovascular outcomes, including sudden cardiac death and HF (Laukkanen et al., 2014).

Findings from prior studies have shown inconsistent results. A recent genetic analysis revealed that the association between liver fat and ASCVD depends on the specific mechanisms by which fat accumulates in the liver (Ahmed et al., 2024). Genetic variants associated with *impaired* hepatic triglyceride export (e.g., *PNPLA3* rs738408 variant) were linked to a reduced risk of coronary artery disease and myocardial infarction, whilst variants associated with *enhanced* de novo lipogenesis were linked to a higher risk of myocardial infarction and coronary artery disease (Ahmed et al., 2024). Another study demonstrated that the *PNPLA3* rs738409 variant may be associated with a reduction in coronary artery disease‐related mortality in men without liver steatosis (Meffert et al., 2018) On the contrary, a Mendelian randomization study, which included the *PNPLA3* rs738409 variant, suggested that genetically defined SLD may be associated with an increased risk of HF, but not coronary artery disease or stroke (Peng et al., 2022). Yet, another recent study found that the *PNPLA3* rs738409 variant was not associated with incident HF (Hydes et al., 2024). The differences in findings may relate to the different cardiac phenotypes assessed (ASCVD versus HF) and the lack of stratification by SLD status in some studies.

The PNPLA3 enzyme is highly expressed in liver and adipose tissue, where it is thought to mediate lipid metabolism (Guo et al., 2025; Zhang et al., 2025). rs738409 induces an isoleucine to methionine protein variant at position 148 (I148M), which may lead to a reduction in the capacity of the PNPLA3 enzyme to breakdown triglycerides, thus promoting liver fat accumulation (Guo et al., 2025; Zhang et al., 2025). Whilst reduced hepatic triglyceride secretion and circulating lipoproteins may theoretically contribute to lower ASCVD risk, the potential effects on myocardial lipotoxicity and risk of HF remain unclear (Butcko et al., 2026). We observed an association between the *PNPLA3* variant and greater LV mass and ventricular volumes in young adults with SLD, but potential mechanisms for this finding cannot be answered by our observational non‐mechanistic study. Interestingly, there were no significant differences in the lipid profile among carriers of *PNPLA3* rs738409C>G compared with non‐carriers. A recent report found that the risk of dyslipidaemia was not higher in individuals with reduced *PNPLA3* expression or following PNPLA3 inhibition (Guo et al., 2025). However, a Mendelian randomization analysis suggested that *PNPLA3* inhibition may increase low‐density lipoprotein cholesterol and total cholesterol levels and increase the risk of ASCVD and HF (Zhang et al., 2025). Given the potential links that have been postulated in the literature, the cardiovascular impact of *PNPLA3* gene variants and its inhibition will need to be determined in further research. This is particularly important since pharmacotherapies to treat liver disease are in development, including antisense oligonucleotides and small‐interfering RNAs to lower PNPLA3 levels (Guo et al., 2025).

Strengths of this study include the utilization of cardiac and liver MRI, enabling comprehensive and accurate assessment. The study also benefits from prospective follow‐up and a well‐characterized cohort with a homogenous age. Limitations include the modest sample size and observational design; therefore, residual confounding is possible and casual relationships between *PNPLA3* rs738409C>G and cardiac remodeling cannot be determined. Moreover, participants were predominantly Caucasian. Analyses stratified by sex, number of G‐alleles or etiology of SLD (e.g., alcohol‐related) were limited by sample size. As this study was hypothesis‐generating in nature, formal adjustment for multiple comparisons was not performed and the findings should therefore be interpreted with caution until validated by further studies. Genotyping and MRI scans were not performed concurrently owing to the longitudinal nature of the study, where assessments were performed across several timepoints. Nonetheless, genotype at age 17 years is likely to reflect genetic exposure at age 27 years.

In conclusion, this observational MRI‐based study of young adults demonstrates a gene‐liver phenotype interaction, whereby the *PNPLA3* rs738409C>G variant may be associated with subclinical changes in cardiac structure and function in the presence of SLD. Whilst causality cannot be determined in this study, the contradictory literature on *PNPLA3* gene variants and cardiac outcomes warrants larger studies to confirm our hypothesis‐generating findings, determine potential underlying pathophysiological mechanisms, and investigate the clinical implications.

## AUTHOR CONTRIBUTIONS

**Nick S. R. Lan:** Conceptualization; formal analysis; investigation; methodology. **Phillip E. Melton:** Conceptualization; data curation; investigation; methodology; resources. **Amro Sehly:** Data curation; investigation; methodology. **Leon A. Adams:** Conceptualization; investigation; methodology; resources. **John K. Olynyk:** Investigation; methodology; resources. **Oyekoya T. Ayonrinde:** Investigation; methodology; resources. **Brendan Adler:** Investigation; resources. **Lawrence J. Beilin:** Investigation; methodology; resources. **Trevor A. Mori:** Conceptualization; data curation; investigation; methodology; project administration; resources; supervision. **Girish Dwivedi:** Conceptualization; investigation; methodology; resources; supervision.

## FUNDING INFORMATION

This work was supported by the NHMRC and the Raine Medical Research Foundation who have provided funding for the Raine Study over the past 30 years. The core management of the Raine Study is funded by The University of Western Australia, Curtin University, The Kids Research Institute Australia, Women and Infants Research Foundation, Edith Cowan University, Murdoch University, The University of Notre Dame Australia and the Western Australian Future Health Research and Innovation Fund [Grant ID WACSOSP2023‐2024]. The 17‐year recall of the Raine Study was supported by an NHMRC Program Grant (Stanley et al., ID 353514); NHMRC (Palmer et al., ID 572613; Beilin et al., ID 403981; Huang et al., ID 1059711); Canadian Institutes of Health Research ‐ CIHR (Lye et al., MOP‐82893). The Pawsey Supercomputing Centre provided computation resources to carry out analyses required with funding from the Australian Government and the Government of Western Australia. The 27‐year recall of the Raine Study was supported by an NHMRC Project Grant (Mori et al., ID 1102106); the Royal Perth Hospital Research Foundation; Heart Foundation Western Australia Branch; Lions Eye Institute; Curtin University; School of Population and Global Health at UWA; Division of Obstetrics and Gynecology at UWA; and Professor John Olynyk. Trevor A Mori was supported by an NHMRC Research Fellowship (1136046).

## CONFLICT OF INTEREST STATEMENT

NSRL has received research funding from Sanofi as part of a Clinical Fellowship in Endocrinology and Diabetes; education support from Amgen, AstraZeneca, Bayer, Boehringer Ingelheim, CSL Seqirus, Eli Lilly, Novartis, Novo Nordisk, and Pfizer; speaker honoraria from Amgen, AstraZeneca, Boehringer Ingelheim, CSL Seqirus, Eli Lilly, Menarini, Novartis, Novo Nordisk, and Sanofi; and has participated in advisory boards for Eli Lilly. AS has received education support from Boston Scientific and Sanofi. LA has received speaking fees from CSL Behring, Novo Nordisk, and Dr. Falk Pharma; grant funding from Novo Nordisk; and has participated in advisory boards for CSL Behring, Novo Nordisk, and Roche Diagnostics. OTA has provided consultancy services to Novo Nordisk and Resonance Health and received speaker honoraria from Norgine, equipment support from Medical Technologies Australia, and Canon Medical Systems ANZ Pty Limited. GD reports paid lectures from AstraZeneca, CSL, Novartis, Novo Nordisk, Pfizer, and Amgen and provides consultancy services and has an equity interest in Artrya Ltd. The remaining authors have no disclosures related to this study. Corporate entities did not have any role in the conduct of this study, analysis or interpretation of data, writing of this manuscript, or the decision to submit it for publication.

## ETHICS STATEMENT

The Raine Study was approved by the University of Western Australia Human Research Ethics Committee, is registered in the Australian New Zealand Clinical Trials Registry (ACTRN12617001599369) and was conducted according to the principles of the Declaration of Helsinki.

## Supporting information


**Table S1.** Baseline characteristics of study population by steatotic liver disease with sex‐stratification.
**Table S2.** Interaction between *PNPLA3* genotype and steatotic liver disease on cardiac remodeling.
**Table S3.** Sensitivity analysis for association between *PNPLA3* genotype and steatotic liver disease on cardiac remodeling, adjusting for body mass index instead of waist circumference.
**Table S4.** Sensitivity analysis for association between *PNPLA3* genotype and steatotic liver disease on cardiac remodeling, adjusting for MRI liver fat fraction, in participants with SLD.
**Table S5.** Descriptive data for cardiac magnetic resonance imaging parameters in those with steatotic liver disease by rs738409C>G in *PNPLA3* carrier status.

## Data Availability

Requests for permission to access the data may be sent to the Raine Study (https://rainestudy.org.au; Email: rainestudy@uwa.edu.au).
