# Supplementary material for: Interplay between PNPLA3 genotype, liver steatosis, and cardiac remodeling in young adults: A magnetic resonance imaging study
Source: Physiol Rep. 2026 Aug 7;14(15):e71040. doi: 10.14814/phy2.71040 (PMC13451627; doi:10.14814/phy2.71040)
Supplement: Supplementary file 1 [file PHY2-14-e71040-s001.docx]

**Supplemental Table 1 – Baseline characteristics of study population by steatotic liver disease with sex-stratification**

| **Characteristic** | **Male** | | **p-value** | **Female** | | **p-value** |
| --- | --- | --- | --- | --- | --- | --- |
|  | **No SLD**  (n=262) | **SLD^a^**  (n=63) |  | **No SLD** (  n=287) | **SLD^a^**  (n=45) |  |
| Height (cm) | 181 (176 - 186) | 182 (177.5 - 187) | 0.345 | 168 (164 - 171) | 167 (163 - 171) | 0.497 |
| Weight (kg) | 80.5 (73 - 88) | 100 (87.5 - 115.5) | **<0.001** | 66 (60 - 74) | 95 (77 - 116) | **<0.001** |
| BMI (kg/m^2^) | 24.3 (22.5 – 26.6) | 30.1 (26.7 - 34.1) | **<0.001** | 23.4 (21.3 - 26.2) | 32.7 (27.1 - 40.5) | **<0.001** |
| BSA (m^2^) | 2.0 (1.9 - 2.1) | 2.3 (2.1 - 2.4) | **<0.001** | 1.8 (1.7 – 1.9) | 2.1 (1.9 - 2.3) | **<0.001** |
| Waist circumference (cm) | 84.0 (79.0 - 90.0) | 101.7 (91.7 - 111.8) | **<0.001** | 75.7 (70.0 - 82.9) | 99.9 (87.3 - 115.2) | **<0.001** |
| Caucasian ethnicity | 250 (95.4%) | 62 (98.4%) | 0.476 | 276 (96.2%) | 43 (95.6%) | 0.692 |
| Paternal cardiac history | 4 (1.7%) | 1 (1.7%) | 0.999 | 1 (0.4%) | 0 | 0.999 |
| Maternal cardiac history | 9 (3.7%) | 0 | 0.214 | 9 (3.4%) | 1 (2.4%) | 0.999 |
| Smoker | 54 (21.8%) | 9 (14.8%) | 0.223 | 39 (13.7%) | 4 (9.1%) | 0.481 |
| Dyslipidaemia | 2 (0.8%) | 1 (1.7%) | 0.487 | 5 (1.8%) | 2 (4.8%) | 0.229 |
| Hypertension^b^ | 2 (0.8%) | 3 (5.1%) | 0.055 | 4 (1.4%) | 4 (9.5%) | **0.012** |
| Systolic BP (mmHg) | 128.2 ± 10.0 | 137.0 ± 12.3 | **<0.001** | 115.7 ± 10.1 | 120.0 ± 13.1 | **0.047** |
| Diastolic BP (mmHg) | 74.2 ± 7.7 | 81.9 ± 7.9 | **<0.001** | 73.9 ± 7.7 | 78.9 ± 9.5 | **<0.001** |
| Diabetes | 3 (1.3%) | 0 | 0.999 | 0 | 0 | - |
| HOMA-IR | 1.0 (0.7 - 1.3) | 2.3 (1.5 - 2.9) | **<0.001** | 1.0 (0.8 - 1.4) | 2.2 (1.7 - 3.4) | **<0.001** |
| Total cholesterol (mmol/L) | 4.7 (4.1 - 5.3) | 5.1 (4.2 - 5.7) | **0.009** | 4.8 (4.3 - 5.4) | 5.0 (4.4 - 5.5) | 0.529 |
| LDL-C (mmol/L) | 2.9 ± 0.7 | 3.2 ± 0.9 | **0.011** | 2.8 ± 0.7 | 3.0 ± 0.7 | **0.017** |
| HDL-C (mmol/L) | 1.3 (1.2 - 1.5) | 1.1 (1.0 - 1.3) | **<0.001** | 1.6 (1.4 - 1.9) | 1.2 (1.1 - 1.3) | **<0.001** |
| Triglycerides (mmol/L) | 0.9 (0.7 - 1.1) | 1.4 (0.9 - 1.9) | **<0.001** | 0.8 (0.6 - 1.1) | 1.2 (0.9 - 1.5) | **<0.001** |
| Alcohol (g/day) | 11.2 (4.5 - 26.1) | 17.5 (2.6 - 32.6) | 0.614 | 4.5 (1.1 - 12.9) | 0.8 (0.2 - 2.9) | **<0.001** |
| Exercise (MET/week) | 2676 (1033 - 4815) | 1440 (480 - 4878) | 0.204 | 1619 (580 - 3477) | 1040 (174 - 3334) | 0.382 |
| MRI liver fat fraction (%) | 1.5 (1.1 - 2.0) | 7.0 (5.3 - 12.0) | - | 1.3 (1.0 - 1.8) | 6.3 (5.2 - 14.1) | - |

a) Diagnosis based on MRI volumetric liver fat fraction >3.55%.
b) Defined as systolic blood pressure >140 mmHg and/or diastolic blood pressure >90 mmHg based on average rested readings.

Data are presented as number (percentage), median (quartile 1 – 3) or mean ± standard deviation. Abbreviations: BMI body mass index; BP blood pressure; BSA body surface area; HDL-C high-density lipoprotein cholesterol; HOMA-IR Homeostatic Model Assessment for Insulin Resistance; LDL-C low-density lipoprotein cholesterol; MET metabolic equivalents; MRI magnetic resonance imaging; SLD steatotic liver disease.

**Supplemental Table 2 – Interaction between *PNPLA3* genotype and steatotic liver disease on cardiac remodelling**

| **Parameter** | **Dominant Model^a^** | | | **Additive Model^a^** | | |
| --- | --- | --- | --- | --- | --- | --- |
|  | **β (95% CI)^b^** | **p-value** | **Interaction p-value for PNPLA3xSLD^c^** | **β (95% CI)^b^** | **p-value** | **Interaction p-value forPNPLA3xSLD^c^** |
| **LV mass index (g/m^2^)** | 0.05 (-0.95 - 1.05) | 0.922 | **0.009** | -0.20 (-0.99 - 0.59) | 0.622 | **0.017** |
| **LV EDVi (ml/m^2^)** | 0.45 (-1.41 - 2.32) | 0.635 | **0.015** | 0.15 (-1.32 - 1.62) | 0.838 | **0.014** |
| LV ESVi (ml/m^2^) | 0.08 (-0.99 - 1.15) | 0.880 | 0.055 | -0.06 (-0.90 - 0.78) | 0.888 | **0.038** |
| LV SVi (ml/m^2^) | 0.37 (-0.87 - 1.61) | 0.559 | 0.052 | 0.21 (-0.76 - 1.19) | 0.668 | 0.065 |
| LV EF (%) | 0.12 (-0.73 - 0.98) | 0.776 | 0.496 | 0.11 (-0.56 - 0.79) | 0.739 | 0.409 |
| LV GLS (%) | -0.30 (-0.71 - 0.12) | 0.162 | 0.966 | -0.33 (-0.66 - 0.00) | 0.050 | 0.682 |
| **RV EDVi (ml/m^2^)** | 0.13 (-2.08 - 2.34) | 0.907 | **0.049** | -0.15 (-1.89 - 1.59) | 0.865 | 0.083 |
| RV SVi (ml/m^2^) | -0.14 (-1.86 - 1.58) | 0.873 | 0.107 | 0.04 (-1.32 - 1.39) | 0.959 | 0.149 |
| RV EF (%) | -0.30 (-1.93 - 1.32) | 0.714 | 0.541 | 0.05 (-1.23 - 1.33) | 0.939 | 0.512 |
| LA volume index (ml/m^2^) | 1.66 (-2.36 - 5.68) | 0.419 | 0.813 | 0.71 (-2.44 - 3.86) | 0.659 | 0.427 |

a) Dominant *PNPLA3* model defined as CC vs CG or GG whilst *PNPLA3* additive model defined as CC vs CG vs GG.
b) Adjusted for sex.
c) Diagnosis based on MRI volumetric liver fat fraction >3.55%.

Abbreviations: CI confidence interval; EDVi end-diastolic volume index; EF ejection fraction; ESVi end-systolic volume index; GLS global longitudinal strain; LV left ventricular; MRI magnetic resonance imaging; *PNPLA3* patatin‐like phospholipase domain‐containing protein 3; RV right ventricular; SLD steatotic liver disease; SVi stroke volume index.

**Supplemental Table 3 – Sensitivity analysis for association between *PNPLA3* genotype and steatotic liver disease on cardiac remodelling, adjusting for body mass index instead of waist circumference^a^**

| **Parameter** | **No SLD**  (n=549) | | **SLD^a^**  (n=108) | |
| --- | --- | --- | --- | --- |
|  | **β (95% CI)^b^** | **p-value** | **β (95% CI)^b^** | **p-value** |
| **Dominant genetic model (CC vs CG/GG)** | | | | |
| **LV mass index (g/m^2^)** | -0.16 (-1.29 - 0.98) | 0.784 | 3.28 (0.72 - 5.83) | **0.013** |
| **LV EDVi (ml/m^2^)** | -0.22 (-2.20 - 1.75) | 0.824 | 6.28 (1.99 - 10.58) | **0.005** |
| **LV ESVi (ml/m^2^)** | -0.41 (-1.60 - 0.77) | 0.491 | 2.12 (-0.88 - 5.10) | 0.161 |
| **LV SVi (ml/m^2^)** | 0.19 (-1.15 - 1.53) | 0.781 | 4.16 (1.38 - 6.94) | **0.004** |
| **RV EDVi (ml/m^2^)** | 1.19 (-3.41 - 1.28) | 0.372 | 6.00 (0.30 - 11.70) | **0.039** |
| **Additive genetic model (0, 1 or 2 copies of the G-allele)** | | | | |
| **LV mass index (g/m^2^)** | -0.33 (-1.26 - 0.60) | 0.489 | 2.02 (0.21 - 3.83) | **0.029** |
| **LV EDVi (ml/m^2^)** | -0.21 (-1.82 - 1.40) | 0.798 | 4.22 (1.20 - 7.25) | **0.007** |
| **LV ESVi (ml/m^2^)** | -0.45 (-1.42 - 0.51) | 0.355 | 1.45 (0.64 - 3.54) | 0.173 |
| **LV SVi (ml/m^2^)** | 0.23 (-0.85 - 1.34) | 0.660 | 2.78 (0.82 - 4.74) | **0.006** |
| **RV EDVi (ml/m^2^)** | -0.86 (-2.77 - 1.04) | 0.374 | 3.22 (-0.82 - 7.26) | 0.117 |

a) Diagnosis based on MRI volumetric liver fat fraction >3.55%.
b) Adjusted for sex, body mass index, hypertension, systolic blood pressure, HOMA-IR and alcohol intake.

Abbreviations: CI confidence interval; EDVi end-diastolic volume index; ESVi end-systolic volume index; HOMA-IR Homeostatic Model Assessment for Insulin Resistance; LV left ventricular; MRI magnetic resonance imaging; *PNPLA3* patatin‐like phospholipase domain‐containing protein 3; RV right ventricular; SLD steatotic liver disease; SVi stroke volume index.

**Supplemental Table 4 – Sensitivity analysis for association between *PNPLA3* genotype and steatotic liver disease on cardiac remodelling, adjusting for MRI liver fat fraction, in participants with SLD^a^**

| **Parameter** | **β (95% CI)^b^** | **p-value** |
| --- | --- | --- |
| **Primary analysis: dominant genetic model (CC vs CG/GG)** | | |
| **LV mass index (g/m^2^)** | 3.07 (0.24 - 5.91) | **0.034** |
| **LV EDVi (ml/m^2^)** | 6.59 (1.90 - 11.27) | **0.006** |
| **LV ESVi (ml/m^2^)** | 2.55 (-0.68 - 5.77) | 0.121 |
| **LV SVi (ml/m^2^)** | 4.04 (1.03 - 7.05) | **0.009** |
| **RV EDVi (ml/m^2^)** | 5.82 (-0.38 - 12.01) | 0.065 |
| **Secondary analysis: additive genetic model (0, 1 or 2 copies of the G-allele)** | | |
| **LV mass index (g/m^2^)** | 1.93 (-0.12 - 3.98) | 0.065 |
| **LV EDVi (ml/m^2^)** | 4.57 (1.19 - 7.94) | **0.009** |
| **LV ESVi (ml/m^2^)** | 1.88 (-0.44 - 4.19) | 0.111 |
| **LV SVi (ml/m^2^)** | 2.69 (0.52 - 4.86) | **0.016** |
| **RV EDVi (ml/m^2^)** | 2.89 (-1.61 - 7.38) | 0.205 |

a) Diagnosis based on MRI volumetric liver fat fraction >3.55%.
b) Adjusted for sex, waist circumference, hypertension, systolic blood pressure, HOMA-IR, alcohol intake and **MRI liver fat fraction (continuous)**.

Abbreviations: CI confidence interval; EDVi end-diastolic volume index; ESVi end-systolic volume index; HOMA-IR Homeostatic Model Assessment for Insulin Resistance; LV left ventricular; MRI magnetic resonance imaging; *PNPLA3* patatin‐like phospholipase domain‐containing protein 3; RV right ventricular; SLD steatotic liver disease; SVi stroke volume index.

**Supplemental Table 5 – Descriptive data for cardiac magnetic resonance imaging parameters in those with steatotic liver disease^a^ by rs738409C>G in *PNPLA3* carrier status**

| **Parameter** | **Male** | | **Female** | |
| --- | --- | --- | --- | --- |
|  | ***PNPLA3* Wild-type Carrier**  (n=40) | ***PNPLA3* rs738409**  **G-allele Carrier**  (n=23) | ***PNPLA3* Wild-type Carrier**  (n=23) | ***PNPLA3* rs738409**  **G-allele Carrier**  (n=22) |
| **LV mass index (g/m^2^)** | 53.2 ± 5.4 | 56.8 ± 8.4 | 39.8 ± 5.1 | 42.1 ± 3.2 |
| **LV EDVi (ml/m^2^)** | 73.3 ± 11.9 | 79.9 ± 11.1 | 63.5 ± 11.5 | 67.5 ± 8.7 |
| **LV ESVi (ml/m^2^)** | 30.9 ± 8.5 | 33.1 ± 6.4 | 24.5 ± 5.3 | 27.0 ± 4.3 |
| **LV SVi (ml/m^2^)** | 42.4 ± 7.2 | 46.8 ± 7.5 | 39.0 ± 7.6 | 40.5 ± 6.5 |
| **RV EDVi (ml/m^2^)** | 87.1 ± 11.6 | 93.9 ± 16.7 | 69.9 ± 11.4 | 72.9 ± 8.1 |

a) Diagnosis based on MRI volumetric liver fat fraction >3.55%.

Data are presented as mean ± standard deviation. Abbreviations: CI confidence interval; EDVi end-diastolic volume index; ESVi end-systolic volume index; LV left ventricular; MRI magnetic resonance imaging; *PNPLA3* patatin‐like phospholipase domain‐containing protein 3; RV right ventricular; SLD steatotic liver disease; SVi stroke volume index.
